# Validation of Chinese Multidimensional Depression Assessment Scale (MDAS) in Inner Mongolia pregnant women and risk factors of antenatal depression in Inner Mongolia in the era of one-child policy

**DOI:** 10.1371/journal.pone.0227944

**Published:** 2020-03-20

**Authors:** Ho Nam Cheung, Stella W. Y. Chan, Joanne M. Williams

**Affiliations:** 1 Department of Social Sciences, School of Arts and Social Sciences, The Open University of Hong Kong, Hong Kong, China; 2 Department of Clinical Psychology, School of Health in Social Science, The University of Edinburgh, Edinburgh, Scotland, United Kingdom; Ben-Gurion University of the Negev Faculty of Health Sciences, ISRAEL

## Abstract

**Background:**

Pregnancy involves physiological changes in reproductive and endocrine systems, and social role changes that can increase the risk of mental health problems. In China, greater emphasis has been given to postpartum depression and its negative impact on infant development. This study examined depression in pregnant women in Inner Mongolia, who are under the influence of cultural values of collectivism and social factors specific to China. Chinese society adheres firmly to traditional values, while market reform, birth-control policy, together with high parental investment in childcare and rearing construct a unique and sometimes unfavorable environment for Chinese women that may influence their depression expression.

**The aims of this study are twofold:**

First, it validated the Chinese Multidimensional Depression Assessment Scale (MDAS), a holistic self-report questionnaire measuring depression severity in four domains of depression-emotional, somatic, cognitive and interpersonal in pregnant women in Inner Mongolia; second, it examined the influences of demographic characteristics (including age, education and employment), pregnancy characteristics (week of gestation, first pregnancy), self-esteem, social support, social activity, work stress, and work-family balance on depression.

**Methods:**

A total of 234 pregnant women, mostly in their third trimester, were recruited in an antenatal hospital in Inner Mongolia and self-reported questionnaires were completed.

**Results:**

Using Confirmatory factor analysis (CFA), MDAS gave rise to a best-fit four-factor model corresponding to each subscale when it was first developed. MDAS also reported high Cronbach’s alpha (0.96) and good convergent validity. Using hierarchical multiple linear regressions with significant demographic variables controlled for, self-esteem, work-family conflict, and social support were found to be significant predictors for depression.

**Conclusions:**

MDAS is a valid scale to be used with Chinese pregnant women, especially in more collectivistic geographical areas. Risk factors specific to the Chinese context add insights to the experience of antenatal depression in China and contribute to understanding depression in from a global mental health perspective.

## Introduction

Pregnancy is a major life event and is also a potentially stressful period of life [1], during which pregnant women adapt to various psycho-social and physiological changes [2, 3], increasing their vulnerability to depression [4]. Growing research evidence in Western societies suggests that depression could begin during pregnancy [5]. For example, Wisner et al. [6] reported that 33% of participants with postnatal depression started to have the disorder during pregnancy. Prevalence rates of antenatal depression have been reported to range between 5% and 41% [7, 8]. Untreated antenatal depression could lead to deteriorating maternal health [9, 10], including greater levels of anxiety [11, 12] and a higher risk for postpartum depression [13, 14]. Antenatal depression is also a risk factor for pregnancy outcomes including a shorter gestation age and complications of pregnancy, such as acute or elective caesarean sections, operative deliveries, perineal tears, excessive bleeding, premature contractions, back pain and preeclampsia [15].

There has been an increasing focus on the importance of mental health screening of pregnant women. The American College of Obstetricians and Gynaecologists has recommended screening for depression at least once during the perinatal period using a standardized, validated tool, with an appropriate follow-up diagnosis and treatment [16]. However, such guidelines have not been introduced in China, despite increasing awareness on antenatal depression.

Although there has been growth in Western research on antenatal maternal mental health, research focusing on Chinese pregnant women is scarce. Yet this is an issue that affects women in China. Prevalence rates of antenatal depression among Chinese women of 4.8% have been reported [17], with 28.5% in South China [18] and 9.9% in Hong Kong [19]. The majority of studies have been conducted in the southern parts of China, such as Shanghai, Guangdong, and Hong Kong, which are believed to have adapted a more individualistic culture. Much less research has been done in the northern parts, such as Inner Mongolia which has been found to be among the most collectivistic areas in China [20].

Schatz [21], in their review of antenatal depression in East Asia (e.g. Taiwan, China, Japan, and Korea), found that Chinese individuals have common risk factors associated with antenatal depression in both Western and Eastern populations, including: age [17, 22], education level [23], occupation, number of miscarriages/abortions [24]. In Asian societies, culture related risk factors, such as premarital pregnancy [25], conflict with in-laws (especially mother in-law) [26], and gender preference [27] have been examined in previous studies. Despite the disadvantaged working condition that pregnant women are facing, the relationship between work stress and antenatal depression has yet to be examined in Mainland Chinese pregnant women. Previous studies such as that by Sanguanklin and McFarlin demonstrate the association between job strain and psychological distress for employed pregnant women in other populations [28]. Some studies [29, 30] also reported that imbalance between work and family life remains the strongest factor linked to mental disorder. All these factors could lead to increasing challenges for Chinese pregnant women in maintaining a work-life balance. In particular, pregnant women are under the combined stress of having less security in employment, the traditional gender role of taking care of the family, pressure as a dual earner, interpersonal stressors in family dynamics, the Chinese birth-control policy, and anxiety about producing a male heir [31].

A methodological barrier to research on antenatal depression among Chinese women has been a lack of validated measures. None of the common measures, despite their good psychometric properties in depression screening, such as the Edinburgh Postnatal Depression Scale (EPDS) [32], General Health Questionnaire (GHQ) [33], Hospital Anxiety and Depression Scale-Depression Subscale (HAS-D) [34], The Patient Health Questionnaire (PHQ) [35] and The Postpartum Depression Screening Scale (PDSS) [36], have been validated on Chinese pregnant women before being put into clinical use with pregnant women. A limited number of validation studies on self-report scales also hinders comprehensive statistical comparison and meta-analysis in the search for the most suitable screening tools for the Chinese pregnant population.

The current study therefore aimed to validate the Multidimensional Depression Assessment Scale (Chinese-MDAS) in a sample of pregnancy women in Inner Mongolia, including the psychometric properties of MDAS and identify the most-fit model of MDAS on Inner Mongolia pregnant women using Confirmatory factor analysis (CFA) following the exploratory factor analysis performed on Inner Mongolia clinical sample in another study. It also aimed to examine risk factors of antenatal depression in this specific cultural context including occupational stress, work-life balance, social functioning, social activities and self-esteem. Demographic, socio-economic, and pregnancy-specific risk factors including age, gestation week, first pregnancy, education attainment, labour market position, and occupation were also investigated. The study aimed to address the following research questions: (1) What is the best-fit model of MDAS on the current sample? (2) Does MDAS demonstrate good psychometric properties? (3) What are the risk factors of antenatal depression on Inner Mongolia pregnant women?

## Materials and methods

### Design

The current research adopted a cross-sectional and quantitative design in assessment of psychometric properties of MDAS and risk factors for depressive symptoms. Multivariate relationships between risk factors and depressive symptoms were examined.

### Sampling and sample

The study recruited a convenience sample at one of the largest maternity and child hospitals in Inner Mongolia. The inclusion criteria were as follows: 1) Patient age between 20 to 40 years; and 2) A full-term pregnancy. The exclusion criteria were as follows: 1) Drug, alcohol or opioid abuse; 2) Considering terminating pregnancy; and 5) Inability to cooperate for any reason.

### Procedure

**Fig 1:**
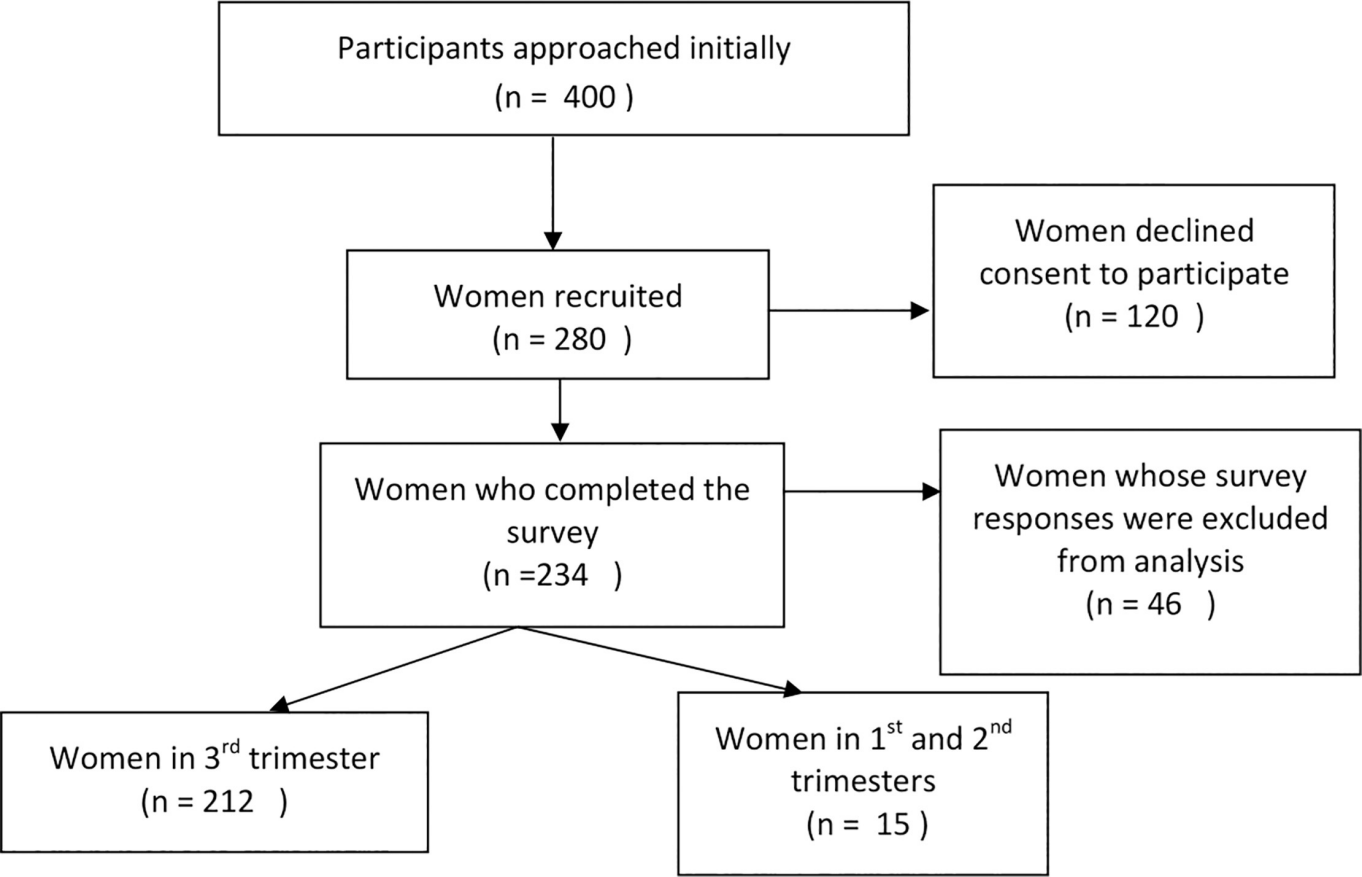
Flow chart of participants recruitment.

Recruitment of pregnant women took place in the waiting room for routine antenatal check-up. A total of 400 pregnant women were approached. Among which 280 agreed to take part in the study while 120 declined. The majority of reasons from declining participation were being uncomfortable in answering depression-related questionnaires, experiencing difficulties in staying focused to complete pages of questionnaires during pregnancy. Among those participants who completed questionnaires but whose data was excluded from analysis: 26 failed to complete a single scale of measure and were excluded, while 20 completed the questionnaire randomly by giving the same answer for every question throughout the questionnaire. (Fig 1) An information pack was handed to all potential participants with a consent form, an information sheet, and a sample questionnaire. Those who agreed to take part in the study were asked to sign the consent form. However, if they wished not to reveal their names their consent was implied by handing in the completed questionnaire anonymously. They were given time to complete the questionnaire while in the waiting room after their check-ups. The researcher was present throughout the administration of the questionnaire to answer any questions. Given the fact that difficulty in concentration is a common consequence during pregnancy, there was no time limit for completion of the questionnaires. The measures were administrated in the following order: depression scales come first, followed by psychosocial measures.

### Measures

The Chinese Multidimensional Depression Assessment Scale (Chinese-MDAS). A 52-item multidimensional depression assessment scale was developed by Cheung and Power [37]. It assesses depressive severity in 4 domains of depressive symptoms: emotional, cognitive, interpersonal, and somatic. Apart from the cognitive subscale, which contains 16 items, all other subscales contain 12 items. A pilot study by Cheung and Power [37] on 100 community participants reported good psychometric properties of the English version, including a high Cronbach's alpha for the whole scale (0.87) and each sub-scale (emotional = 0.87; cognitive = 0.88; somatic = 0.83; interpersonal = 0.89). A significant high positive correlation (0.77) between the new scale and BDI-II indicated a good convergent validity. It was translated into Chinese using the parallel back-translation procedure [38] and found to have psychometric properties on Inner Mongolia clinically depressed patients with Cronbach’s alpha above 0.9. The total scale and each subscales also had a high and significant correlation with BDI (r = 0.72; p < .001) (Cheung, Williams, & Chan, under review).

#### Beck Depression Inventory (BDI)

The BDI is a 21-item self-rating scale developed for the assessment of depression in the psychiatric population [39]. Salamero, Marcos [40] reported a Cronbach’s alpha of 0.85. A cut-off value greater than 16 gave rise to high sensitivity (0.83) and specificity (0.89). The scale has been widely studied and translated into multiple languages, including Chinese. The Chinese version of the scale has been shown to have good psychometric properties [41]. Zhang, Wang [42] validated the BDI on 335 Chinese participants and reported a split-half validity of the scale of 0.879 and Cronbach’s Alpha as 0.89. The current study therefore used the first version, and the Cronbach’s alpha co-efficient was 0.85 on this sample. Despite the fact that the BDI-II has widely replaced the original version, many studies still use the first version, due to its availability in China and long history of validation in the Chinese population. The current study therefore used BDI-I due to ease of access and its established use with Chinese populations. The Cronbach’s alpha co-efficient (N = 234) for the BDI in this sample was 0.85.

#### Edinburgh Postnatal Depression Scale (EPDS)

The Edinburgh Postnatal Depression Scale (EPDS) is a 10-item self-rating scale developed by Cox and colleagues [32]. The EPDS is one of the most widely used self-rating scales during pregnancy around the world [1]. In Chinese population, Guo et al. [43] validated the Chinese version of EPDS on 732 women at 3–14 days postpartum. This study reported a Cronbach’s alpha of 0.76 and Spearman’s co-efficient ranged from 0.37 to 0.67. Guo, Wang [44] reported an optimal cut-off value of the EPDS as 9.5 in second and third trimester women, giving rise to sensitivity and specificity of 0.79 and 0.83 respectively. In the present study (N = 234), the Cronbach’s alpha was 0.83.

#### Sheehan’s Disability Scale (SDS)

The Sheehan’s Disability Scale (SDS) is a 3-item self-report instrument measures impairments from the domains of work, family life/home responsibilities and social/leisure activities [45]. It has been tested with adequate reliability(0.79–0.91 95% Confidence Interval) [46] and validity [46–48]. The Chinese version has also been validated with adequate psychometric performances with intra-class correlation coefficient 0.89 (0.82–0.93 95% CI) and Cronbach's alpha 0.9 [49]. In the present study (N = 234), the Cronbach’s alpha was 0.84.

Rosenberg Self-Esteem Scale (SES). The Rosenberg Self-Esteem Scale (SES) [50] is a 10-item scale rated on a 4-point Likert scale from strongly agree to strongly disagree. It has been validated in the Chinese language and has widely been used in Chinese society in research into self-esteem [51]. In the present study (N = 234), the Cronbach’s alpha was 0.71.

#### Work Stress Survey (WSS)

The work stress survey is a 10-item brief self-rating questionnaire [52] assessing job strain, effort-reward imbalance and work-family conflicts, which are shown to be associated with depression [53]. It demonstrated an adequate Cronbach’s alpha of 0. 74 in the current sample (N = 234).

For later analysis of the components of work stress associated with antenatal depression, exploratory factor analysis (EFA) was performed on WSS with an Oblimin (oblique) rotation. It gave rise to a three-factor model which accounted for 41.35% of the total variance in items. Factor 1 (Interpersonal-related pressure) accounted for 28.17% of total variance. Factor 2 (Personal responsibilities) accounted for 8.6% of variance, whereas the last factor (Working environment) accounted for 4.58% of the total variance (Appendix 1).

#### Work-Family Conflict (WFC) and Family-Work Conflict Scale (FWC)

Developed by Netemeyer, Boles [54], the measure includes the 5-item Work-Family conflict (WFC) scale and the 5-item Family-Work conflict scale (FWC) designed to measure the conflict and balance between work and family life. WFC measures the inter-role conflict between work and family in which the general demands of time devoted to, and strain created by the job hinders individuals from carrying out family-related responsibilities, while FWC measures conflicts in the reverse direction. These two scales were reported to have an average Cronbach’s alpha of 0.88 for WFC and 0.86 for FWC across three samples [54]. A Cronbach’s alpha of 0.86 was found for the WFC and of 0.91 for the FWC in the present sample (N = 234).

#### Social Functioning Scale-subscale (SFS subscale)

The Social Functioning Scale (SFS) was developed by Birchwood et al. [55] with the original intention of capturing social performance and functioning in schizophrenia patients. In this study a subscale was used which consists of 22 social events. Participants rated the frequency of their taking part in the activities on a four-point scale from ‘Never’ to ‘Often’. Adequate validity, reliability and sensitivity were found for both clinical and community samples. Birchwood et al. [55] documented the reliability as 0.80 for the full scale and 0.82 for the social activity subscale. The Cronbach’s alpha for the current sample was 0.83.

EFA (N = 234) was performed on SFS subscale using oblique rotation yielded a two-factor solution, which accounted for 55.4% of the total variance in items (factor 1 eigenvalue = 10.29; factor 2 eigenvalue = 1.89). Factor 1 (Interpersonal-orientated activities) accounted for 46.79% of variance. Factor 2 (Individual-orientated activities) accounted for 8.61% of variance (Appendix 2)

#### The Significant Others Scale Short Version (SOS)

The SOS was developed by Power and Champion [56] to measure the perceived quality of individuals’ most important relationships. Participants were required to name up to 6 significant others, and rate the level of perceived emotional and practical support from each of the 6 people on a 7-point scale (1 = never to 7 = always). The original scale was reported to have a good six-month test-retest reliability (0.73–0.83) [56]. In the current study, a short 4-item SOS was scored in terms of total emotional support and practical support perceived by the participants from summing up the difference of ideal rating and actual rating indicating the level of support. In the current sample, the SOS scale was found to have a Cronbach’s alpha of 0.82 for emotional support and 0.72 for practical support.

### Statistical analyses

To identify the best fitting factor model of MDAS on pregnant women, confirmatory factor analysis was conducted to compare the model fit of three models: a 3-factor model (affective, interpersonal, somatic) was previously established on an Inner Mongolia clinically depressed sample using exploratory analysis (Cheung, Williams, & Chan, under review), a 4-factor model based on the original design of the questionnaire, and a 1-factor model using Mplus version 7 [57]. The robust weighted least square with mean and variance correction estimator (WLSMV) was adopted to test model fit. The estimator gives a robust result when the outcome is categorical and violates multivariate normality. It is also most appropriate in the case of a modest sample size. The model fit of the three non-nested models were compared of their fit indices. These included the comparative fit index (CFI); Bentler’s [58] study criterion of .90 or higher, and the root mean squared error of approximation (RMSEA); of 0.08 or lower [59–61] whereas RMSEA below 0.06 indicated a good fit [62].

To test the psychometric properties of the MDAS with the best-fit model, statistical package SPSS (version 20.0) was used to perform statistical analyses normality of the data, depression prevalence and assessing the psychometric properties of the MDAS including internal consistency indexed by Cronbach’s alpha (α) [63] and item-total correlations, and construct validity assessed by Spearman correlation between the MDAS, BDI and EPDS.

Investigation on the risk factors of antenatal depression included five hierarchical regression models which were conducted to determine the variables significantly contributing to the overall depressive severity and severity of depressive symptoms in four dimensions (i.e., emotional, cognitive, somatic and interpersonal subscales) on the MDAS. For each regression model, the same set of independent variables were included: the total scores of SES, WSS, WFS, FWS, subscale scale of SFS and the emotional and physical subscales of SOS. Categorical variables (qualification, employment, and occupation) were recoded into dummy variables. Exploratory factor analysis (EFA) with an Oblimin (oblique) rotation was conducted on WSS and SFS subscale and the factor scores were entered into regression models to determine the factor that contribute to increasing risk of antenatal depression. Demographic variables were entered into the first block of predictors while the psychosocial variables were entered into the second block. Assumptions were checked including Cook’s distances (>1.0) [64], tolerance (<0.10) or the variance inflation factor (VIF >10) of each independent variable [65], Spearman’s correlation (>0.7) between the independent variables, the Durbin–Watson statistic (1.50 to 2.50), and normal distribution of standardized residuals. Log-transformations of the dependent variables including total score of MDAS, and the scores of each subscale were performed as they did not fulfil the assumptions of linear regression. Each of five regression models was examined by investigating several criteria: the overall model fit was indicated by the value of *R*^2^ and the value of *F* ratio and its associated *p* value. All statistical tests were two-tailed and at a significance level of 0.05. Upon the establishment of the initial model with all demographic and psychosocial variables entered into the model, the least significant variables were removed from the model one at a time. A new model was then fitted without the least significant variable. The step was repeated until a resulting model was obtained containing only significant predictors. For each subsequent model fitted, ANOVA was checked for its significance.

## Results

### Demographic characteristics of participants

A total of 234 pregnant women with a mean gestation week of 35.56 (S.D. = 5.88) were recruited to the study. The mean age was 29.13 years (S.D. = 3.26), in a range between 22 and 40 years old. The sample characteristics are shown in Table 1. The majority of participants were in their first pregnancy (79%) and third trimester (93.4%) of pregnancy.

**Table 1:** Descriptive statistics of pregnant sample.

Demographic Variables	N (%)	Demographic Variables	N (%)
**Trimester**		**Occupation**	
First	8 (3.5)	Professional	121 (52.6)
Second	7 (3.1)	Administrative	46 (20.0)
Third	212 (93.4)	Service	45 (19.6)
**Qualification**		Craftsmanship	1 (0.4)
Middle School	13 (5.6)	Labour (physically demanding manual work)	5 (2.2)
High School	27 (11.5)	Others	12 (5.2)
Undergraduate	176 (75.2)	**Medical history**	
Postgraduate	18 (7.7)	History of depression	18 (7.7)
**Employment**		Currently under Medication	8 (3.4)
Full-time employment	193 (82.5)		
Full-time student	3 (1.3)		
Unemployed	16 (6.8)		
Others	22 (9.4)		

### The best-fit model of MDAS on pregnant women

Thirty-six percent of women scored above 10 on the EPDS, indicating that they were in a higher risk group for developing antenatal depressive symptoms. Possible reasons for the higher prevalence could be attributed to inflated scale score due to the presence of somatic symptoms. In this study, the majority of participants were in their third trimester (>90%) and might experience many somatic symptoms due to their pregnancies.

The CFA model of the 3-factor structure failed to support the three-factor structure due to two highly correlated factors of factor 2 (Interpersonal) and 3 (Somatic). As a remedy, factor 2 and 3 were combined into a new factor to eliminate this problem. Hence the resulting 2-factor model (Affective, Interpersonal-somatic) was compared in this section with the hypothetical 4-factor model in the original design of MDAS and the 1-factor model.

The CFA model fit (Table 2) of the three models shows significant chi square differences (p<0.01), suggesting that all three models have unexplained variances. However, it is unrealistic to anticipate a perfect-fit model between hypothesized and observed data [65]. In addition, statistically significant χ^2^ difference could also be the result of sample size and small variations in the data. Hence, the root mean square error of approximation (RMSEA) has been proposed to be a more realistic fit index than chi-square statistics [65]. All of the models showed similarly good indices of CFI and TFI (>0.95). However, statistics of the RMSEA indicated that the 4-factor model and 1-factor model provide a better fit to the current data (RMSEA < .08) and the 2-factor model showed a less good fit (RMSEA close to 0.1). In support of a 4-factor model as the best-fit model, the next section presents the results of the psychometric properties of the original 4-factor model of MDAS including the whole MDAS scale and each subscale (Cognitive, Emotional, Somatic, Interpersonal).

**Table 2:** Confirmatory factor analysis model fit indices.

Fit indices	4 factor model (Cognitive, emotional, somatic, interpersonal)	2 factor model (Interpersonal and somatic)	1 factor model (Depression)
Chi square,	3864.03	3887.44	5075.17
(df)	1268	776	1274
P	0.00	0.00	0.00
RMSEA,	0.068	0.095	0.082
(90% CI),	0.065–0.070	0.092–0.098	0.080–0.084
P RMSEA < = 0.05	0.00	0.00	0.00
CFI	0.97	0.95	0.95
TFI	0.96	0.95	0.95

### Psychometric properties of the Chinese-MDAS

#### Internal consistency and group discrimination

The overall Cronbach’s alpha for the whole MDAS was .96, Cronbach’s alpha estimates for the four subscales of the Chinese-MDAS ranged from .84 to .93 (Emotional, α = .93; Cognitive, α = .91; Somatic, α = .85; Interpersonal, α = .84). Based on the criterion of .30 as an acceptable corrected item–total correlation [63], only one item (item 35) showed an item-total correlation lower than the criterion. However, it was retained due to its importance in content for further analysis.

#### Convergent validity

Spearman correlations showed significant positive and moderate correlations (r) in a range from .44 to .59 between the MDAS, each subscale, the EPDS and the BDI. As expected, the MDAS and all the four subscales showed a significant positive and moderate correlation with the BDI and EPDS. The correlation (r = 0.28, p<0.01) between the MDAS and Sheehan’s Disability Scale was significant but less robust. The convergent validity represents the correlation between the New Multidimensional Depression Scale with the EPDS and BDI (Table 3).

**Table 3:** Spearman correlation matrix of MDAS, BDI and EPDS.

Measures	BDI	EPDS
MDAS Total score	0.59**	0.56**
Emotional Subscale	0.44**	0.54**
Cognitive subscale	0.54**	0.58**
Somatic subscale	0.52**	0.37**
Interpersonal subscale	0.47**	0.42**

**p<0.001

### Risk factors for antenatal depression

Results of the five regression models were summarized in Table 4. Model 1 was significant (F (5, 215) = 14.257, p< 0.001) with an R^2^ of .249. The second model, which examined the association between total score of the emotional subscale and the independent variables, was significant (F (3, 217) = 15.262, p< 0.001) with an R^2^ of .174. The third model examining the association between total score of the cognitive subscale and the independent variables was significant (F (4, 216) = 16.659, p< 0.001), with an R^2^ of.236. The fourth model with total score of the somatic subscale as dependent variable, a significant equation was found (F (3, 219) = 10.847, p< 0.001) with an R^2^ of .129. The total score of the interpersonal subscale as dependent variable reported a significant equation (F (3, 227) = 12.755, p< 0.001), with an R^2^ of .144. Table 4 showed the unstandardized B and the standardized β for individual predictors in each model.

**Table 4:** Summary of hierarchical regression analysis for variables predicting total and each subscale of MDAS.

Variables	MDAS total scale			Emotional subscale			Cognitive subscale			Somatic subscale			Interpersonal subscale		
	B	S.E.B	β	B	S.E.B	β	B	S.E.B	β	B	S.E.B	β	B	S.E.B	β
**Postgraduate**	.143	.054	.160**				.145	.060	.146*				.156	.065	.151*
**Full time employment**	-.094	.038	-.150*							-.101	.046	-.139*	-.119	.045	-.163**
**Occupation Laborious**				.290	.124	.144*									
**SES**	-.022	.004	-.349**	-.020	.005	-.254**	-.027	.004	-.391**	-.018	.005	-.245**	-.023	.005	-.323**
**WFS**	.008	.004	.133*				.009	.004	.126*						
**SOS emotional subscale**	.057	.018	.190**				.065	.020	.193**						
**SOS Physical subscale**				.096	.022	.268**				.052	.022	.154*			

*p<0.05

**p < .01

## Discussion

Using the EPDS cut-off value of 10, a prevalence rate for prenatal depressive symptoms of 36% was found among this sample of pregnant women from Inner Mongolia. Similar prevalence rates have been reported in other studies in Chinese populations. For example, Li and colleagues ] reported that 39% of women scored above 9.5 on the EPDS to indicate signifying depression, among whom 36.4% were in their first trimester, 40.2% were in their second trimester and 39.8% were in their third trimester. By contrast, a much lower prevalence rate of 6.4% was recorded by Lee et al. [66] using structured clinical interviews for DSM-IV (SCID-NP). Several reasons could explain the high prevalence rate: Firstly, a cutoff value of EPDS was never developed for Inner Mongolia pregnant women, so the current cutoff value from literature could give rise to an inflated rate of prevalence; secondly, lifestyle and hormonal changes towards labor may have contributed to elevated prenatal negative affect [67]. In addition, one cannot rule out the possibility of a high risk of antenatal depression in Inner Mongolia that should be addressed in future studies. Finally, a higher prevalence has also been reported using self-report measures in comparison to clinically diagnostic tools. However, although MDAS could give rise to false positives due to its inclusion of somatic symptoms, its holistic approach in presenting symptoms in various dimensions allows the opportunity to clarify the profile of symptoms across scores in each subscale.

### Comparison of factor models of the MDAS for pregnant women in Inner Mongolia

CFA supported that the originally designed 4-factor model had better model fit indices than the 3-factor model developed from the clinically depressed sample in Inner Mongolia clinical sample (established in Cheung et al.) or a 1-factor model. This result supported using the total score of the MDAS in Inner Mongolian pregnant women to indicate severity of depression and using the 4 subscales to assess depressive symptoms in each dimension. The results led to important clinical implication of measuring severity in individual dimensions and studying the risk factors associated with them. It also supported that the somatic subscale, which has been the centre of controversy for depression screening in pregnant women, should be reconsidered before being ruled out from self-report instruments. In fact, researchers such as Nylen and Williamson [68] opposed removing somatic symptoms in self-report instruments when assessing antenatal depression as they argued that it could lead to reduced scale validity as the assessment does not fully reflect how severe pregnant women’s mood disturbance is. In addition, inclusion of an interpersonal subscale, which fits the cultural characteristics of Chinese populations, is also supported.

### Assessing antenatal depression with the Chinese-MDAS

Following the result of a best-fit 4-factor model of the MDAS, the psychometric characteristics of the Chinese-MDAS points to a possibility of adopting a multidimensional symptomatic scale for pregnant samples in Inner Mongolia and other parts of China. In particular, a high internal consistency was found for the Chinese-MDAS and each subscale. A significant positive correlation was also found between the Chinese-MDAS, BDI and EPDS, suggesting a good convergent validity of the new scale with standardized and well-studied scales. A significant correlation between the MDAS and the SDS suggested its ability in indicating impairment through the measurement of interpersonal symptoms that in SDS are indicators of the level of severity of depression.

### Risk factors of antenatal depression in Inner Mongolia

Results of this study shielded light on societal-related risk factors in Chinese pregnant women. In line with previous studies unemployment was identified as a risk factor for antenatal depression. Full time employment, by contrast, was negatively associated with overall depressive severity as well as the somatic and interpersonal aspects of depression. Greater job security and less financial strain have both been demonstrated to negatively relate to depressive symptoms [69]. This finding could also suggest that their protective functions against antenatal depression. However, women with higher education attainment were found to be more likely to have increased antenatal depressive symptoms than those with middle school education. This finding contradicts previous findings that a lower level of education is a risk factor for depression as it links to financial difficulties [70]. A possible explanation is related to greater responsibility participants shoulder in jobs that associated with their higher educational attainment and the consequent pressure of being both breadwinners and family carers in modern Chinese societies. In contrast to the mediating effect of work stress (effort-report imbalance and low control) on occupational class and status and depressive symptoms reported by Hoven and Wahrendorf [71], all occupational categories did not contribute significantly in associating with antenatal depression except for physically demanding laborious job, which is significantly related to depressive severity on the emotional subscale.

In terms of psychosocial risk factors, self-esteem emerged as a significant predictor of depressive symptoms. This finding is in line with previous findings [72] and argued for the importance of social support as a protective factor against depression in pregnant women and its direct and moderating effects on women’s perceived stress and antenatal depressive symptoms [73]. The present findings also added to the existing research that not only does the overall perception of level of social support affect depressive severity [74], but also the discrepancy of perception between expected and actual supported mattered to depression in various domains during pregnancy. As Lee [75] postulated, given how much Chinese culture values pregnancy as providing a continuation of the family lineage, and under the one-child policy, the foetus is greatly cherished. This study pointed to the importance of emotional support for Inner Mongolian pregnant women during pregnancy for prenatal care. This is also an under-researched topic in Inner Mongolia.

Work-family conflict was found to be a significant risk factor for overall severity in depression during pregnancy as well as emotional and cognitive domains of depressive symptoms. This could suggest the difficulties to balance work and family could be a source of stress for Chinese women in their later stage of pregnancy. This result is consistent with previous proposals for financial and family struggle for Chinese women, who are still likely to remain in their jobs till the third trimester. This struggle thus is linked to antenatal depression in this empirical study. The finding shields light into the adverse situation that Chinese women could be facing in pregnancy and points to directions of future policymaking towards improved quality of life and mental health during pregnancy. As the majority of participants were in their third trimester (>90%), it is also worth investigating these job-related factors such as work stress, work-life balance and depression on participants in early pregnancy. Naturally, the non-significant association found between the social activities and depression severity is likely that participants in their third trimesters could be highly restrained to home activities, contributing to a less obvious social effect. Future studies on pregnant women in their earlier stages of pregnancy could be conducted to examine a more apparent effect of social activities on antenatal depression.

### Limitations

This study has several limitations. First, test-retest reliability of the MDAS was not established in the current study due to mobility of pregnant women between various hospitals. In other words the same group of recruited participants would possibly switch to another hospital, imposing difficulties for a retest. The issue should be addressed in future studies when there is more time and resources. In future studies a group of depressed pregnant women could be recruited to test the power of MDAS to distinguish depressed and non-depressed pregnant women in China with the aid of standardized diagnostic interviews second; more cultural-related risk factors relevant to China could be brought in to future studies to examine interaction effects between factors. Lastly, demographic variables measured were based on participants’ retrospective recalls, which could subject to bias. Future studies could employ official medical record for a more objective measures of demographic factors.

## Conclusions

A 4-four factor model (emotional, cognitive, somatic, and interpersonal) was identified to provide the best fit for capturing the depressive symptoms in pregnant women in Inner Mongolia. The MDAS demonstrated good psychometric properties in terms of reliability, validity and dimensionality. A combination of employment and qualification, low self-esteem, work-family conflict and social support were found to be associated with the total score and various subscales of the MDAS. When controlling for demographic variables, low self-esteem, work-family conflict and social support were significantly associated with depressive symptoms in general and in different domains. The results shed light on the use of MDAS to assess depression severity in Chinese population and adds information about Chinese cultural-specific risk factors to current literature on depression.
